# Prevalence of *Helicobacter Pylori* in Obese Adults: A Literature Review

**DOI:** 10.33549/physiolres.935753

**Published:** 2025-12-01

**Authors:** Adéla NOVOTNÁ, Miloš CHUDÝ, Nikol GOTTFRIEDOVÁ, Daniel KARAS, Pavol HOLÉCZY, Marek BUŽGA

**Affiliations:** 1 Department of Epidemiology and Public Health, Faculty of Medicine, University of Ostrava, Ostrava, Czech Republic; 2 Department of Physiology and Pathophysiology, Faculty of Medicine, University of Ostrava, Ostrava, Czech Republic; 3 Department of Neurology, University Hospital, Ostrava, Czech Republic; 4 Institute of Laboratory Medicine, Faculty of Medicine, University of Ostrava, Ostrava, Czech Republic; 5 Department of Surgery, Vítkovice Hospital, Ostrava, Czech Republic; 6 Department of Surgical Disciplines, Faculty of Medicine, University of Ostrava, Ostrava, Czech Republic; 7 Department of Human Movement Studies, Faculty of Education, University of Ostrava, Ostrava, Czech Republic; 8 Institute of Laboratory Medicine, University Hospital, Ostrava, Czech Republic

**Keywords:** Obesity, BMI, *Helicobacter pylori*, Prevalence

## Abstract

Obesity is a major health challenge of the 21^st^ century and the number of obese people is increasing worldwide and with it the number of people suffering from obesity-related diseases. The relationship between the presence of obesity and *Helicobacter pylori (H. pylori)* infection has long been a subject of interest across the literature. The presented review aimed to analyze the prevalence of *H. pylori* in adults with higher BMI. A literature search was conducted using the electronic databases Scopus, PubMed and Web of science. The term *“Helicobacter pylori”* was searched with obesity-related keyword combinations. A total of 1.109 records, published in the last 18 years, were identified through the database search. Of these articles, seven were ultimately included in the analysis. Although the studies did not all agree on the same conclusion, most of them have shown that the greater prevalence of *H. pylori* can be observed in participants with higher BMI, than in normal-weight individuals. A higher prevalence of *H. pylori* can be observed in obese individuals. However, further research is needed to clearly confirm the BMI-*H. pylori* relationship.

## Introduction

Obesity is a major disease of the 21^st^ century and its prevalence is increasing in all regions of the world. Between 1980 and 2013, obesity increased globally from 28.8 % to 36.9 % in men and from 29.8 % to 38 % in women, which is alarming as obesity brings along many comorbidities [1]. However, no country has so far succeeded in reversing this pandemic disease. Obesity is part of a global syndrome in which universal societal factors such as the obesity pandemic, the malnutrition pandemic and climate change occur and interact [2,3].

*Helicobacter pylori* infection has a phenomenon of collective aggregation and represents a major societal problem. *Helicobacter pylori (H. pylori)* is a gram-negative curved bacteria that adheres to the gastric mucosa and the interstitial space [4]. In 1994, the World Health Organization (WHO) classified *H. pylori* as a established human carcinogen therefore classified as a Group 1 carcinogen. Current recommendations indicate that *H. pylori* eradication is indicated in all adults with proven active infection, but routine screening of asymptomatic individuals in the general population is not recommended [5]. Infection can cause stomach diseases such as peptic ulcer disease and gastritis, and approximately 1 % of *H. pylori*-positive patients develop gastric cancer [6].

*H. pylori* affects a significant proportion of the global population [7]. According to O’Connor *et al*., more than 50 % of the population worldwide has been infected with this bacteria, according to Muhammad *et al*., the global estimated prevalence is around 40–50 % [4,8]. As mentioned, *H. pylori* infection is strongly associated with gastric ulcers and gastric cancer, and its eradication represents a highly effective method to reduce the incidence and mortality of stomach cancer [9]. A study by Liu *et al*., focused on risk factors for *H. pylori* infection, identified age, higher total cholesterol level, lower albumin level, higher low density lipid (LDL) cholesterol level and higher fasting blood sugar level as factors increasing the chance of infection [10].

The results of a large study by Suki *et al*., show strongly significant differences in the prevalence of *H pylori* infection in relation to body mass index (BMI) [11]. According to the study by Kamada *et al*., the correlation between the presence of *H. pylori* and higher-degree obesity is refuted with the claim that individuals with *H. pylori* start gaining weight only after undergoing *H. pylori* eradication [12]. The study by Wu *et al*., showed an inverse relationship between higher BMI and the presence of *H. pylori* [13]. The study by Thjodleifsson *et al*., suggests that *H. pylori* only accompanies insulin resistance and obesity, while not being related to systemic inflammation [14,15]. Therefore, the association between a higher degree of obesity (or BMI) and the presence of *H. pylori* in the human body is still considered a controversial topic [16].

This article aims (1) to summarize the knowledge on the prevalence of *H. pylori* in the obese individuals compared to normal-weight population, and to organize the information gained in this area over the last 20 years. Furthermore, we aim (2) to provide readers with the prevalence of *H. pylori* in various worldwide.

## Materials and Methods

### Search strategy

A comprehensive literature search was conducted in the electronic databases Scopus, PubMed, and Web of Science between October 30, 2023, and December 23, 2023. The search strategy combined terms related to *Helicobacter pylori* and body mass index (BMI). Representative search terms included: [*“relation between BMI and Helicobacter pylori,” “obesity and Helicobacter pylori,”* and *“Helicobacter pylori epidemiology in world populations.”*].

### Inclusion criteria

Studies were eligible for inclusion if they met the following criteria: (1) Contained predefined keywords related to *H. pylori* and BMI; (2) Investigated the prevalence of *H. pylori* infection in relation to BMI in human participants; (3) Were original research articles published between 2005 and 2023; (4) Were written in English.

### Exclusion criteria

The following records were excluded: (1) Duplicates retrieved from multiple databases; (2) Non-original works such as book chapters, brochures, or conference proceedings; (3) Studies not directly focused on the prevalence of *H. pylori* in relation to BMI; (4) Studies that did not clearly describe the diagnostic method for *H. pylori* detection or lacked sufficient methodological detail; (5) Animal studies.

## Results

**Fig. 1:**
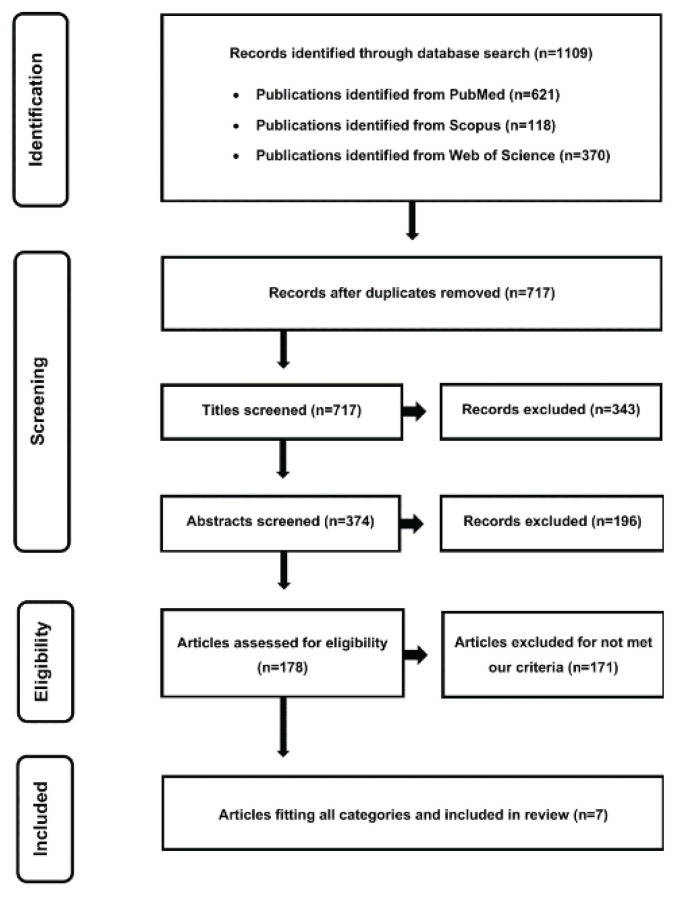
Flow diagram.

The initial search identified 1109 records. After removal of duplicates (n=392), 717 unique records remained. Of these, 710 were excluded for not meeting the eligibility criteria, leaving seven articles for final inclusion in the analysis. The study selection process is summarized in Figure 1 (flow diagram).

All seven included studies aimed to investigate the prevalence of *H. pylori* in overweight/obese and normal-weight people in various regions of the world. Further details of the included studies are shown in Table 1. The oldest study was published in 2005, the most recent in 2022. The studies differed in the number of respondents, with the smallest cohort consisting of 214 individuals (61.2 % male) and the largest of 235107 individuals (35.9 % male). They also differed in the detection method used to determine the presence of *H. pylori* infection, with four authors using Enzyme-Linked Immunosorbent Assay (ELISA), two using urea breath test (UBT) and one using endoscopy.

The studies did not have the same criteria for selecting obese participants between them, but 71.4 % of the studies included individuals with a BMI>30. Among these studies, the most commonly used method for the detection of *H. pylori* was the Enzyme-Linked Immunosorbent Assay (ELISA) method, which represents a simple, sensitive, rapid, reliable and versatile test for quantification of antigens and antibodies, and is done from blood serum. It is useful due to the extreme distinguishing ability of antibodies to recognize an almost infinite number of antigenic structures [17]. The second most used method across included studies was the urea breath test (UBT). The UBT is a non-invasive method of detecting *H. pylori* with high specificity and sensitivity, and is performed from exhaled air. However, the specificity of this test is decreased if other urease-producing bacteria are present in the intestine of the subject [18]. Endoscopic examination/Endoscopic resection, used in one study, allows the collection of a representative sample for definitive histopathological evaluation, which may also refine the diagnosis, including the assessment of the depth of invasion [19]. The study by Suki *et al*., with the largest cohort consisted of 32824 subjects with higher BMI, revealed the strong association between *H. pylori* infection and BMI, while the prevalence in obese subjects was 65 % [11]. The study by Arslan *et al*., had the smallest cohort with 103 participants with higher BMI, and the prevalence in obese subjects was 57.2 % [16].

**Fig. 2:**
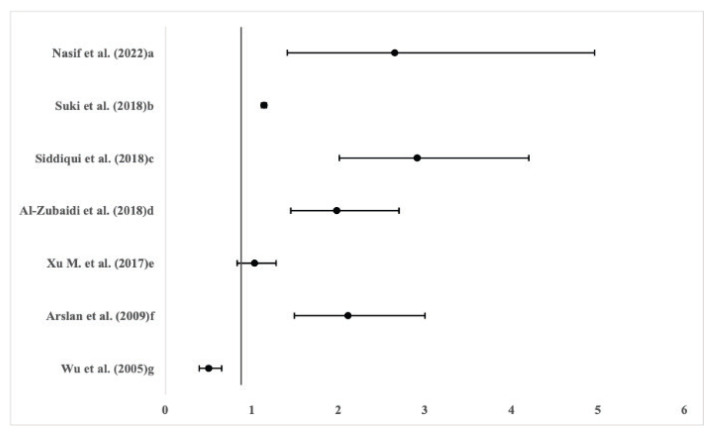
Forest plot showing odds ratio in relationship between higher BMI and prevalence of *H. pylori* in comparison with normal weighted subjects; Inclusion criteria: a, d, f, g – BMI≥30, b – BMI≥30–35, c – BMI>23.1, e – BMI≥28.

In all of these studies, the study population was predominantly from countries located on the Asian continent, so we do not have sufficient information to compare this trend in, e.g., Europe. The selected studies compared a study group of subjects with normal BMI (normal-weight individuals) with a group with higher BMI, and investigated the possible difference between the obese and normal-weight individuals. The values at Figure 2 below represent the odds ratios of seven selected studies and describe the relationship between the prevalence of *H. pylori* infection and higher BMI.

The average prevalence in the entire study cohorts was 55.17±7.09 %. The minimum prevalence was 41.5 % and the maximum was 62.40 % [16,26]. The mean prevalence in the obese groups was 63.66±13.11 %, with minimum prevalence of 43.7 % and maximum 80.60 % [13,20]. According to the conclusions of the 71.4 % included studies, the prevalence of *H. pylori* is significantly higher in the obese individuals compared to those with normal weight, and to general population. A different conclusion was found in a study by Xu *et al*., which observed no significant difference between these groups [21].

The oldest study by Wu *et al*. reported completely different conclusions. In this study, authors found an inverse relationship between obese and normal-weight participants, i.e., suggested that obesity may act as a protective factor for the *H. pylori* infection [13].

The method used to detect *H. pylori* varied across studies, however, it did not play a primary significant role in the difference between groups. Another factor that may have influenced the results, was the nationality of the study population. Studies in Chinese, Israeli, Turkish, Pakistani, Saudi Arabian and Taiwanese populations have shown different results in the association of *H. pylori* between overweight/obese and normal-weights individuals, as well as in prevalence of general population.

Although the authors found very interesting results, research in this area is still insufficient. Further in-depth research is needed to better understand the BMI-*HP* relationship.

## Discussion

*Helicobacter pylori* remains a widespread infection with significant clinical consequences, including its well-known role in chronic gastritis, peptic ulcers, and gastric cancer. Although the World Health Organization classifies *H. pylori* as a Group 1 carcinogen, the relationship between *H. pylori* infection and body mass index (BMI) is complex and not yet fully understood.

The seven studies reviewed here present fairly consistent findings regarding the association between BMI and *H. pylori* prevalence. Early studies, such as Wu *et al*., suggested a potential protective effect of higher BMI against *H. pylori* infection. However, the year of publication (2005) of this study may play a role, as the other included studies were published in time period 2009–2022. Another factor possibly affecting the findings of this one study, may be that it is not reported, whether the study subjects had already undergone eradication for *H. pylori* before the study [13]. More recent research, including Suki *et al*., reported a significant positive association, suggesting that obesity may increase the risk of *H. pylori* infection. These conflicting results are likely due to differences in study design, diagnostic methods (including but not limited to ELISA, urea breath tests, and endoscopy), and varying population characteristics, such as age, geographic region, diet, and socioeconomic factors. A systematic review and meta-analysis by Hooi *et al*. that looked at the prevalence of *H. pylori* in 62 countries revealed that there are huge differences in the prevalence of this bacteria between countries [23]. The prevalence of *H. pylori* ranged from 18.9 % (Switzerland) to 87.7 % (Nigeria), and based on prevalence estimates, it was found that approximately 4.4 billion people were infected with *H. pylori* in 2015, meaning that more than half of the world’s population was infected with *H. pylori* [23]. A systematic review by Peleteiro *et al*. including studies from 1968–2013 looked at the prevalence of *H. pylori* in 22 countries – 5 countries from South and North America, 6 countries from Asia, 10 countries from Europe and 1 country from Oceania (Australia) [24]. It notes that the prevalence is generally higher in countries in North/South America and Asia, especially compared to Europe [24]. For example study by Bureš et. al. showed that the overall prevalence of *H. pylori* infection in the czech population is 23.5 %. The overall prevalence of *H. pylori* in the Czech Republic, central Europe, has declined significantly over the past 10 years, from 41.7 % in 2001 to 23.5 % in 2011 [28]. At the same time, the prevalence was found to be almost twice as high in countries with a high incidence of stomach cancer. According to this review, there is also a possible correlation between the age of individuals and *H. pylori* infection, with the risk of infection increasing with increasing age [24].

In addition, the association with *H. pylori* may be mediated by the presence of metabolic comorbidities, such as dyslipidemia, insulin resistance, and hypertension, which are frequently observed in individuals with obesity. This complex interaction makes it difficult to establish a direct cause-and-effect relationship and emphasizes the importance of using analytical methods that account for these possible confounding factors.

Regional differences in *H. pylori* prevalence further highlight the influence of environmental and lifestyle factors. For example, prevalence may be affected by genetic predispositions or region-specific dietary habits. Socioeconomic status and living conditions also modify the risk of exposure, as reflected by the globally observed differences in prevalence between developing and developed countries.

Given the inconsistencies and limitations of current research, future studies should adopt standardized diagnostic criteria and methodologies to improve comparability. Moreover, multinational cohort studies with comprehensive data on metabolic health, environmental exposures, and genetic factors are needed to clarify the relationship between BMI and *H. pylori* and to inform targeted prevention and treatment strategies.

## Conclusions

The prevalence of *Helicobacter pylori* infection varies considerably by country, region, and socioeconomic status, with developing countries generally exhibiting higher rates than developed ones. Whether obesity is an independent risk factor for *H. pylori* infection remains unclear. Most studies suggest that individuals with higher BMI tend to have a higher prevalence compared to normal-weight individuals, although results are not fully consistent. These inconsistencies may be influenced by differences in study design and population characteristics. Further research using standardized diagnostic methods and diverse populations is necessary to clarify the relationship between BMI and *H. pylori* infection and its clinical significance.

## Figures and Tables

**Table 1:** Overview of the included studies.

*Author, Year*	Country	Number of participants (% men)	Overall prevalence of *H. pylori* among all participants in %	Number of obese participants (% men)	Prevalence of *H. pylori* among obese individuals [%]		Mean age in all participants ± SD	Detection method	Findings
*Nasif et al. 2022*	Saudi Arabia	298 (40.90)	62.40 %	69 (sex unspecified)		78.30 %	47.17±9.27	ELISA	High BMI is associated with *H. pylori* infection
*Al-Zubai et al. 2018*	Saudi Arabia	640 (65.40)	57.90 %	340 (66.5)		66.20 %	Obese: 31.54±8.27 Controls: 30.90±7.93	Endoscopy	High BMI is associated with *H. pylori* infection
*Siddiqui et al. 2018*	Pakistan	698 (53)	57.20 %	258 (sex unspecified)		80.60 %	44±16	UBT	High BMI is associated with *H. pylori* infection
*Xu et al. 2017*	China	3039 (91.8)	53.90 %	388 (sex unspecified)		54.60 %	49±19	ELISA	No association between high BMI and *H. pylori* infection
*Suki et al. 2017*	Israel	235 107 (35.9)	61.42 %	32 824 (sex unspecified)		65.00 %	49±18.1	UBT	High BMI is associated with *H. pylori* infection
*Arslan et al. 2009*	Turkey	214 (61.2)	41.50 %	103 (68.92)		57.20 %	Obese: 24.3±5.4 Controls: 25.5±5.4	ELISA	High BMI is associated with *H. pylori* infection
*Wu et al. 2005*	Taiwan	1097 (41.2)	51.86 %	414 (27.8)		43.70 %	31.9±9.2	ELISA	High

BMI is protective factor for *H. pylori* infection

SD=Standard deviation; ELISA=Enzyme-Linked Immunosorbent Assay; UBT=urea breath test; BMI=Body Mass Index.

## Abbreviations

BMI: Body Mass Index
e.g.: For example
ELISA: Enzyme-Linked Immunosorbent Assay
HDL: High density lipid
i.e.: That is
*H. pylori*: *Helicobacter pylori*
LDL: Low density lipid
UBT: Urea Breath Test
WHO: World Health Organisation
